# Case Report: Staged multidisciplinary nursing management of grade 4 oral mucositis after pediatric hematopoietic stem cell transplantation

**DOI:** 10.3389/fped.2026.1897148

**Published:** 2026-07-27

**Authors:** Shanshan Chen, Yan Lin, Zeli Ma, Yajie Yang, Zhuoting Yuan, Xuan Liu, Jie You, Huiying Xu, Ting Chen

**Affiliations:** 1 Department of Stomatology, Nanfang Hospital, Southern Medical University, Guangzhou, China; 2 Department of Pediatrics, Nanfang Hospital, Southern Medical University, Guangzhou, China

**Keywords:** case report, hematopoietic stem cell transplantation, oral mucositis, pediatric nursing, *α*-thalassemia

## Abstract

Severe oral mucositis (OM) after pediatric hematopoietic stem cell transplantation (HSCT) becomes especially challenging when complicated by pancytopenia, platelet transfusion refractoriness, bleeding, and high nutritional risk, which also make oral care hard to deliver. We report a 13-year-old girl with transfusion-dependent *α*-thalassemia who developed OM on day +6 after unrelated-donor HSCT and progressed to World Health Organization grade 4 on day +17, with severe ulceration, pseudomembrane, trismus, inability to eat, bleeding, and anxiety. The case focuses on how a staged, multidisciplinary nursing pathway kept essential oral and supportive care feasible and safe through continuous assessment, phase-specific intervention, and team coordination. Behavioral techniques (Tell–Show–Do, music and animation distraction, caregiver-assisted care) enabled mouth rinsing and ultraviolet (UV) treatment despite severe pain. OM improved to grade 1 at discharge (day +32) and grade 0 at day +84; pain fell from a Numeric Rating Scale (NRS) score of 8 to 0, anxiety from a Self-Rating Anxiety Scale (SAS) score of 56 to 39, and nutritional risk from a Pediatric Yorkhill Malnutrition Score (PYMS) of 4 to 2 by discharge, with no fatal bleeding, sepsis, catheter-related infection, or recurrence. Because many interventions were delivered concurrently, recovery cannot be attributed to any single measure; the staged pathway was temporally associated with clinical improvement and may have supported recovery.

## 1 Introduction

Oral mucositis (OM) is a common complication of hematopoietic stem cell transplantation (HSCT), occurring in up to 80% of pediatric recipients and often peaking during neutropenia (1). Severe OM (WHO grade ≥3) may cause uncontrolled pain, interruption of oral intake, and infection, thereby prolonging hospitalization and complicating post-transplant recovery (2). The mucosal injury arises from conditioning regimen cytotoxicity, which disrupts epithelial renewal and compromises local immune defenses (3). Basic oral care, photobiomodulation, and cryotherapy are supported as preventive strategies (4) but are often insufficient once severe OM is established, particularly in pediatric patients who have lower pain thresholds, reduced treatment cooperation, and heightened anxiety (5, 6). We report a 13-year-old girl with transfusion-dependent *α*-thalassemia who developed WHO grade 4 OM after unrelated-donor HSCT, complicated by profound neutropenia, refractory alloimmune thrombocytopenia, gastrointestinal bleeding, nutritional vulnerability, swallowing dysfunction, and anxiety. This case presents a staged multidisciplinary nursing pathway that kept essential oral and supportive care feasible and safe when these complications occurred together, with explicit reasoning for why each intervention was chosen, when it was introduced, and how it was adjusted.

## 2 Case presentation

### 2.1 Patient information

A 13-year-old girl had intermediate *α*-thalassemia at 9 months with 12 years of regular transfusions and iron chelation therapy. Both parents were carriers of the *α*-thalassemia trait. She had no known drug allergies, and her growth and development were age appropriate. On admission (day −14): height 155 cm, weight 41.0 kg, body mass index 17.1 kg/m^2^ (healthy-weight range, ∼25th–50th percentile for age). Baseline oral health showed suboptimal hygiene with plaque and multiple caries but no recurrent ulceration or oral infection. A pre-transplant dental program (October 27–31, 2023) was provided including restorations, pit-and-fissure sealing, supragingival scaling, fluoride varnish, and oral hygiene education, and pre-transplant examination confirmed intact mucosa without active caries or periodontal disease. The conditioning regimen combined anti-thymocyte globulin (5 mg/kg total), busulfan (3.2 mg/kg/day ×3), fludarabine (40 mg/m^2^/day ×5), thiotepa (410 mg total), and cyclophosphamide (15 mg/kg/day on days −8/−7 and 50 mg/kg/day on days +3/+4). Stem cells were infused on January 8, 2024 (day 0; 20 × 10⁸/kg, CD34 + 2.072 × 10⁶/kg) and again on day +1 (7.0 × 10⁸/kg, CD34 + 0.65 × 10⁶/kg) for a low CD34 + count.

### 2.2 Clinical findings

On day +6, the patient developed gingival erythema, small oral ulcers, sore throat, and mild diarrhea. OM then progressed and by day +14–16 there was hemorrhagic crusting of the lower lip, diffuse erosions of palate, gingiva, and tongue, pseudomembrane formation, and spontaneous bleeding from ulcer surfaces. Mouth opening was limited to two fingerbreadths. On day +17, she was unable to tolerate oral intake and developed hematemesis; oral intake was withheld (water allowance) and anticoagulation was discontinued. Pain intensity peaked at Numeric Rating Scale (NRS) 8. The patient exhibited fear of pain and bleeding, with difficulty sleeping (SAS 56).

**Figure 1:**
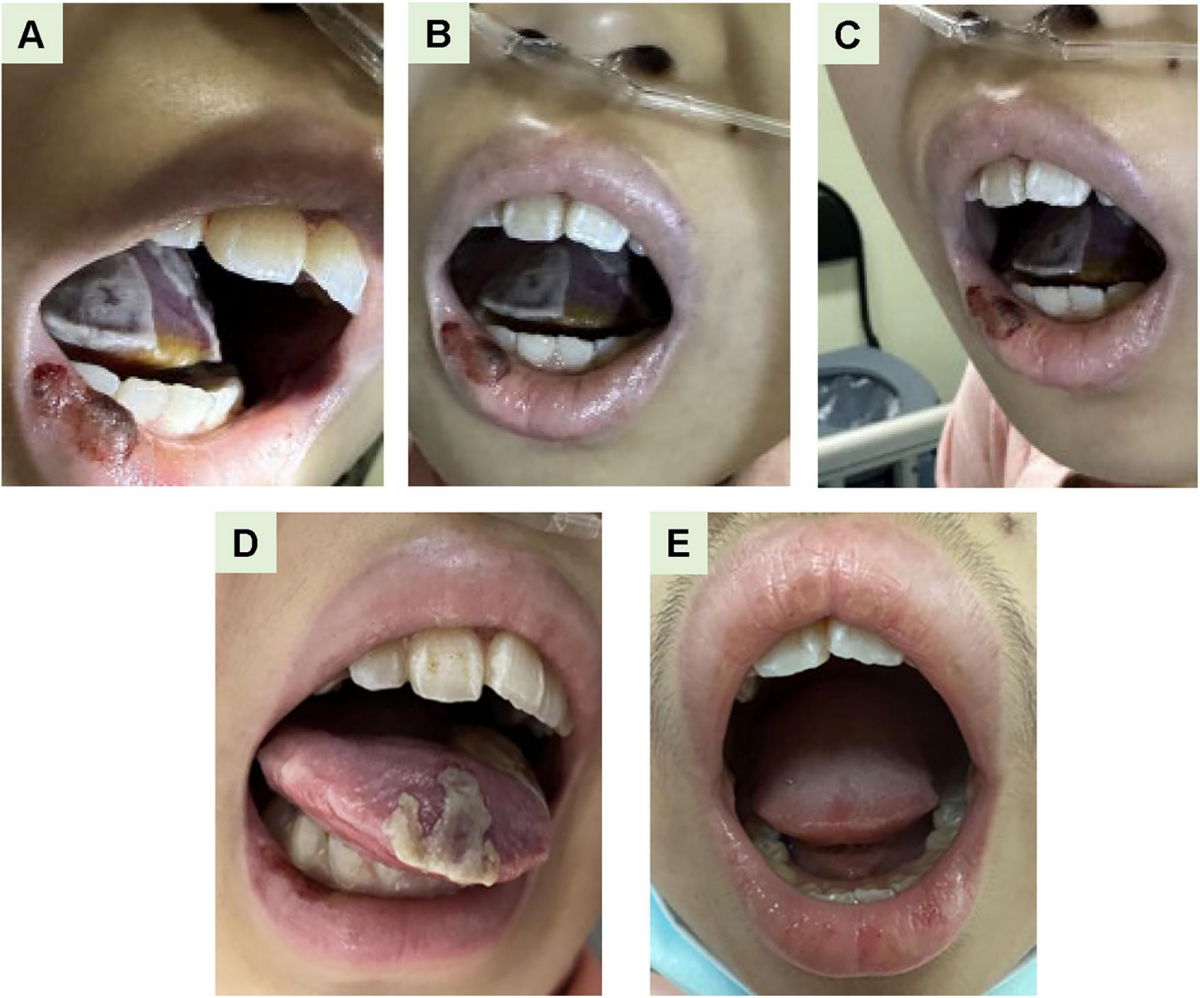
Clinical manifestations of oral mucositis in the pediatric patient at different stages. **(A–C)** January 25, 2024 (D + 17); **(D)** February 1, 2024 (D + 24); **(E)** April 1, 2024 (D + 84).

Pancytopenia was present (white blood cell 0.01 × 10⁹/L, hemoglobin 44 g/L, platelet 9 × 10⁹/L); platelet transfusion refractoriness was attributed to anti-platelet antibodies (GPIIb/IIIa+, GPIa/IIa+, and GMP140+). Fever occurred with elevated C-reactive protein (CRP), procalcitonin (PCT), and interleukin-6. Nutritional risk was high (PYMS 4); albumin was 38.1 g/L (day −6/+4), rose to 44.3 g/L (day +14), then fell to 38.1 g/L by discharge. Swallowing dysfunction (EAT-10 37) and moderate dependence (Barthel 50) reflected pain, feeding difficulty, fatigue, and bed rest. Representative oral findings at the peak, early repair, and follow-up stages are shown in Figure 1. The clinical course is summarized in Table 1.

**Table 1:** Clinical course and outcomes.

Transplant Day	OM grade/clinical course	Key management	Outcome
Before D0	Baseline oral vulnerability (suboptimal hygiene, plaque/pigment, multiple caries) but no recurrent ulcers; mucosa intact before HSCT.	Pre-transplant complete oral treatment.	No active caries, periodontal disease, or mucosal injury before transplant.
D0	Stem cell infusion; entry into a high-risk period for mucosal injury, cytopenia, infection, bleeding, and nutritional deterioration.	Routine oral surveillance, infection prevention, and supportive care.	Defined as the first infusion day.
D + 6 to +7	OM grade 1 during marrow suppression.	Intensified oral assessment; gentle hygiene, mucosal protection, and local supportive care.	OM identified early.
D + 8 to +11	OM grade 2; increasing discomfort and mucosal lesions.	Reinforced mouth-care adherence, pain observation, infection surveillance, and family-assisted care.	Progressive phase; close monitoring.
D + 12 to +16	OM grade 3; worsening pain, lesions, and reduced oral intake.	Analgesic care, mucosal protection, nutritional assessment, and bleeding/infection monitoring.	Deterioration risk; crisis-phase preparation.
D + 17 to +18	OM grade 4; severe pain, pseudomembrane, hemorrhagic crusting, trismus, no oral intake, mucosal and gastrointestinal bleeding, pancytopenia, nutritional interruption, anxiety.	Mucosal rest, parenteral nutrition, analgesia, hemostatic care, antimicrobial/antifungal therapy, close assessment, and psychological support.	Peak severity; no fatal bleeding or sepsis.
D + 19 to +23	OM grade 3; pain, fever, and lesions began improving though severe injury persisted.	Crisis-phase measures continued and adjusted by pain, bleeding, infection indicators, and oral tolerance.	Early repair phase.
D + 24 to +27	OM grade 2; bleeding and pain decreased; oral intake gradually resumed.	Diet advanced cautiously by mucosal healing, pain, swallowing tolerance, and gastrointestinal symptoms.	Oral intake and cooperation improved.
D + 28 to +32	OM grade 1 before discharge.	Gentle hygiene, nutritional transition, discharge education, and caregiver training.	Discharged on D + 32 with a home-care plan.
D + 84	Complete oral mucosal healing (outpatient).	Face-to-face and caregiver assessment of recurrence, pain, diet, and hygiene.	OM grade 0; no recurrent ulceration or bleeding.

## 3 Diagnostic assessment

### 3.1 Diagnosis and grade 4 basis

WHO grade 4 OM was confirmed by a stomatologist on day +17 because of confluent multi-subsite ulceration, extensive pseudomembrane, spontaneous bleeding with hemorrhagic crusting, trismus (two fingerbreadths), and complete inability to tolerate oral intake. Pain, nutrition, anxiety, functional status, and swallowing were assessed using the NRS, PYMS, SAS, Barthel Index, and EAT-10, respectively. Laboratory monitoring included blood counts, CRP/PCT/interleukin-6, albumin, electrolytes, and blood cultures during febrile episodes.

### 3.2 Etiology

The OM was multifactorial. Onset during deep aplasia after intensified conditioning, with rapid progression while white blood cells were 0.01 × 10⁹/L and platelets 1–3 × 10⁹/L, indicated that direct mucosal toxicity from the busulfan/thiotepa/cyclophosphamide regimen was the most plausible initial driver. Profound neutropenia and immunosuppression probably delayed repair and increased infection susceptibility; refractory thrombocytopenia aggravated the hemorrhagic phenotype; and swallowing dysfunction and undernutrition further impaired epithelial repair.

### 3.3 Differential diagnosis and infection assessment

Herpes simplex virus stomatitis was less likely because there were no vesicles or perioral crusting; oral lesion swab PCR was not performed because thrombocytopenia limited mucosal sampling. Oral candidiasis was also less likely because the pseudomembrane was hemorrhagic-fibrinous rather than curd-like, and no local fungal culture or smear confirmation was available. Bacterial superinfection was unconfirmed because oral secretion cultures did not proceed and blood cultures remained negative—which were not considered sufficient to exclude local infection. Oral graft-vs.-host disease (GVHD) was lower-probability given early onset during neutropenia without lichenoid or extra-oral features. Infection was tracked through temperature, CRP/PCT/IL-6, blood cultures, and oral appearance. Empirical broad-spectrum antibacterial and antifungal therapy was adjusted by the pediatric team according to fever, inflammatory markers, blood counts, and clinical course. Serial serologic monitoring of cytomegalovirus, Epstein–Barr virus, herpes simplex virus, and human herpesvirus 6 testing was negative; peripheral-blood targeted next-generation sequencing detected human herpesvirus 7 (6 reads) on day +24, interpreted as low-level and of uncertain local significance because fever, ulceration, and PCT were already improving, so no antiviral was added.

## 4 Therapeutic intervention

Nursing care followed three dynamically adjusted phases (Table 2), coordinated with physician-directed treatment by a multidisciplinary team. Stomatology graded OM and directed local care; pediatrics managed hematology disease and general condition; nutrition planned diet; gynecology suppressed menstruation (oral progesterone 100 mg twice daily) for bleeding risk; psychology supported coping; and proctology, ophthalmology, and otolaryngology screened for occult infection (none found).

**Table 2:** Staged nursing pathway.

Phase	Assessment criteria	Nursing goals	Core interventions	Monitoring and transition
Phase 1: Early progressive (D + 6 to +11; grade 1–2)	Early OM with mucosal erythema/ulceration, increasing discomfort, preserved but vulnerable oral-care tolerance.	Detect progression early; maintain cleanliness as tolerated; reduce pain and anxiety; improve cooperation.	Frequent assessment; gentle mouth care and rinse education; mucosal protection; caregiver involvement before procedures.	OM grade, lesion area, pain, bleeding, rinse tolerance, fever, intake, cooperation. Advance if grade 3, worse pain, lower intake, bleeding, or systemic deterioration.
Phase 2: Severe/peak (D + 12 to +18; grade 3–4)	Severe pain, reduced/interrupted intake, pseudomembrane or hemorrhagic crusting, trismus, mucosal/GI bleeding, infection not fully excluded.	Prevent complications; control pain and bleeding; protect mucosa; maintain nutrition; deliver essential oral care safely.	Minimized stimulation; analgesia; hemostatics; parenteral nutrition; antimicrobials; atraumatic assessment; caregiver and psychological support.	Pain, respiratory/sedation status, bleeding, fever, blood counts, nutrition, electrolytes, lesions. Advance when ≤ grade 3, less bleeding, better pain, and signs of repair.
Phase 3: Early repair and transition phase (D + 19 to +32; grade 3→1)	OM decreasing from grade 3 to 1; pain and bleeding improving; intake resumed; caregiver able to assist home observation.	Promote repair; advance diet safely; prevent recurrence/infection; restore self-care; prepare for home care.	rhEGF after bleeding subsided; diet advanced liquid → semi-liquid → regular; gentle hygiene; discharge education and warning-sign training.	OM grade, pain, intake, weight, swallowing, bleeding, fever, recurrence, bleeding, adherence. Endpoint: complete healing, pain resolution, no recurrence (confirmed D + 84).

Oral care, behavioral management, and UV. Because pain and friability made brushing and rinsing difficult, a Tell–Show–Do approach with music/animation distraction and reassurance enabled rinsing and UV treatment; when brushing was intolerable, a stomatologist taught atraumatic plaque removal with a moistened cotton swab, and caregivers assisted. UV irradiation (254 nm; 20 s/session) targeted the lesions once or twice daily from day +8, with non-target areas shielded and no hot or acidic intake for 30 min afterward; only mild dryness occurred.

Mouth-rinse and pH. Oral pH was checked about every 8 h as an adjunctive indicator. Saline was used for routine cleaning, sodium bicarbonate by clinical assessment for an acidic environment, Kangfuxin liquid (a traditional Chinese medicine preparation used locally in China for mucosal repair) throughout for repair, and chlorhexidine only during grade 3–4 OM (days +14–23) for prevention of infection.

Pain and opioid. Sore throat began on day +6; tetracaine rinses were used on days +8–10; pain peaked at NRS 8 around day +17. A fentanyl transdermal patch (25 µg/h) for 72 h was prescribed by the treating physicians after multidisciplinary assessment because severe oral pain was not adequately controlled by local anesthetic rinses and non-opioid analgesics. Given the patient's young age and lack of previous opioid exposure, nurses performed enhanced monitoring of respiratory rate, consciousness, sedation, gastrointestinal symptoms, and skin reactions, with naloxone available at bedside. No respiratory depression or excessive sedation occurred. 2% lidocaine pre-meal rinses (days +19–27) facilitated eating. Pain gradually improved and resolved on day +27.

Hemostasis and bleeding-risk nursing. With platelet refractoriness (3 × 10⁹/L on day +8; 1 × 10⁹/L on day +12), a critical-illness notice, bed rest, and trauma avoidance were instituted. Hemostasis of ulcer was conducted with norepinephrine bitartrate injection (2 mL applied to irrigate the wound surface twice daily with concurrent saliva suction) on days +20 and +21. Nursing prioritized oral/pharyngeal, gastrointestinal and transfusion-response monitoring; platelets recovered to 40, 53, and 61 × 10⁹/L by days +27, +30, and +32.

Nutrition and rhEGF. During mucosal rest, physician-directed total parenteral nutrition (three-in-one) provided approximately 1,200–1,500 kcal/day (∼30–37 kcal/kg) with amino acids ∼1.2–1.5 g/kg; refeeding was monitored with serial potassium, phosphate, and magnesium, identifying and correcting recurrent hypokalemia. Diet advanced by mucosal healing, pain, swallowing, and gastrointestinal symptoms: water allowance (days +17–18), liquid (day +20), semi-liquid (day +24), and regular (day +28). Recombinant human epidermal growth factor (rhEGF) spray (∼200 IU/spray once daily) was applied to lesions after evening cleaning until healing, which was introduced after bleeding subsided reflecting bleeding risk, tolerance, and staged goals.

## 5 Follow-up and outcomes

Because of immunosuppression and parental concern, the patient stayed mainly homebound until the day +84 visit; nursing surveillance used telephone follow-up every 2 weeks and parental reporting of pain, intake, sleep, fever, mucosal appearance, adherence, and mood. Serial outcomes are shown in Table 3. OM improved to grade 1 at discharge and grade 0 at day +84; pain fell from NRS 8 to 0, anxiety from SAS 56 to 39, and nutritional risk from PYMS 4 to 2.

**Table 3:** Serial outcome measures at key time points.

Measure	Day +6 (onset)	Day +17 (peak)	Discharge (Day +32)	Day +84
WHO OM grade	1	4	1	0
Pain (NRS)	2	8	1	0
Nutritional risk (PYMS)	4	4	2	0
Anxiety (SAS)	–	56	39	–
Swallowing (EAT-10)	–	37	Improving	Normal
Functional status (Barthel)	–	50	Improving	Near-independent
Body weight (kg)	39.7	39.1	36.2	41.3
Platelet count (×10⁹/L)	12	9	61	–
Major complications	Mild diarrhea	GI bleeding; pancytopenia; platelet refractoriness	Resolving	None

## 6 Discussion

This case illustrates the nursing complexity of WHO grade 4 OM after pediatric unrelated-donor HSCT, in which routine oral care was limited by severe pain, trismus, oral and gastrointestinal bleeding, profound pancytopenia, platelet transfusion refractoriness, nutritional interruption, and anxiety. Rather than demonstrating any single novel intervention, its value lies in operationalizing supportive care during a high-risk pediatric crisis: a staged pathway linking repeated assessment to phase-specific goals and escalation triggers, multidisciplinary input, caregiver-assisted atraumatic oral care, nutrition and bleeding-risk management, and safety monitoring for high-risk supportive treatments. OM improved from grade 4 (day +17) to grade 1 at discharge and grade 0 at day +84, but concurrent medical therapy and hematologic recovery preclude causal attribution from a single case.

The etiology was multifactorial, with conditioning toxicity as the likely initiator and neutropenia, immunosuppression, bleeding, and undernutrition as aggravators, while infection and oral GVHD were possible but unconfirmed. The day +17 peak was later than the typical days +8–11 in multicenter pediatric cohorts (7), consistent with these cumulative factors, and supports vigilant oral assessment beyond the first 2 weeks in high-risk children. The principal strength was phase-specific adaptation: escalation during progression, hemostasis and mucosal rest at the peak, repair measures after bleeding subsided, and post-discharge surveillance, with clarified nursing contributions across assessment, monitoring, education, caregiver training, and coordination.

Limitations include the single-case design; unimplemented microbiology, biopsy, salivary analysis, and complete viral testing; use of the SAS rather than pediatric-specific anxiety tools; and concurrent interventions that prevent attribution. UV, Kangfuxin liquid and rhEGF may be unavailable or unapproved elsewhere, and the timing of rhEGF administration was determined by the department's empirical practice. These observations need confirmation in larger studies.

## 7 Patient and caregiver perspective

The patient found severe oral pain, bleeding, and inability to eat or drink the most distressing experiences and initially resisted rinsing and inspection for fear of worsening them. Repeated explanation, stepwise analgesia, music distraction, and her mother's participation in gentle oral care helped her cooperate. Her mother reported that structured discharge instructions and telephone follow-up helped the family recognize warning signs, adjust diet, and regain confidence in home care.

## Data Availability

The original contributions presented in the study are included in the article/Supplementary Material, further inquiries can be directed to the corresponding authors.
